# Acaricidal Activity of Liquid Culture Filtrates from Nematophagous Fungi Against *Rhipicephalus microplus* Ticks

**DOI:** 10.3390/pathogens15070772

**Published:** 2026-07-22

**Authors:** Oscar Guadalupe Barrón-Bravo, Pedro Mendoza-de Gives, César Andrés Ángel-Sahagún, Ricardo Avilés-Ruiz, Rubén D. Garza-Cedillo, José L. Arispe-Vázquez, Juan Patishtan-Pérez, Gustavo Pérez-Anzúrez

**Affiliations:** 1 Instituto Nacional de Investigaciones Forestales, Agrícolas y Pecuarias, Centro de Investigación Regional Noreste, Campo Experimental Las Huastecas, Carretera Tampico-Mante Km 55, Villa Cuauhtémoc, Altamira 89610, Tamaulipas, Mexico; barron.oscar@inifap.gob.mx (O.G.B.-B.); aviles.ricardo@inifap.gob.mx (R.A.-R.); patishtan.juan@inifap.gob.mx (J.P.-P.); 2 Centro Nacional de Investigación Disciplinaria en Salud Animal e Inocuidad, INIFAP, Boulevard Paseo Cuauhnahuac No. 8534, Col. Progreso, Jiutepec 52556, Morelos, Mexico; tavopzaz@gmail.com; 3 Departamento de Medicina Veterinaria y Zootecnia, DICIVA, CIS, Universidad de Guanajuato, Irapuato 36821, Guanajuato, Mexico; csahagun@ugto.mx; 4 Campo Experimental Río Bravo, CIRNE-INIFAP, Río Bravo 88900, Tamaulipas, Mexico; garza.ruben@inifap.gob.mx; 5 Campo Experimental Iguala, CIRPAS, INIFAP, Iguala 40000, Guerrero, Mexico; arispe.jose@inifap.gob.mx

**Keywords:** filamentous fungi, ixodicides, parasites, ticks, nematophagous fungi

## Abstract

*Rhipicephalus microplus* ticks harm cattle health and productivity. Frequent use of ixodicides can result in resistant tick populations and environmental contamination, as residues enter soil. Nematophagous (NF) and entomopathogenic fungi (EF) are natural enemies of nematodes and insect pests. This study tested the in vitro lethal effects of liquid culture filtrates (LCFs) from selected NF on *R. microplus*. Fungi were grown in Czapek-Dox broth for 30 days at room temperature to obtain LCFs, which were tested using the immersion method. Experimental groups included: (1) *Arthrobotrys musiformis*, (2) *Purpureocillium lilacinum*, (3) *Flavocillium subprimulinum*, (4) *Lecanicillium psalliotae*, (5) ixodicide mixture (permethrin and chlorpyrifos), (6) negative control (water), and (7) negative control (Czapek-Dox broth). Differences in tick mortality among filtrate treatments starting on day 10 were observed. Filtrates from *L. psalliotae* and *A. musiformis* caused the highest mortality, with *A. musiformis* reaching 40% and *L. psalliotae* 33.3% on days 14, 16, and 18 (*p* < 0.01). We anticipated that combining ixodicides would lead to substantial tick mortality; however, the ixodicidal compounds (chlorpyrifos and permethrin) resulted in 33.3% mortality by day 18, which was similar to that observed for *L. psalliotae* during the same period. Filtrates from *P. lilacinum* and *F. subprimulinum* had no effect. The ixodicide treatment led to a significant reduction in oviposition, averaging 69.8% (*p* < 0.05). LCFs of selected NF could be used against either nematodes or *R. microplus* ticks.

## 1. Introduction

The cattle tick is a major parasite that impacts the health and productivity of cattle and serves as a vector for diseases such as Anaplasmosis and Babesiosis [1]. While chemical ixodicides can help reduce tick populations and lessen the spread of diseases associated with these parasites, they come with significant drawbacks.

Parasitism is one of the biggest problems in animal production, with a significant economic impact. Therefore, chemical control methods have become less effective due to parasite resistance. Consequently, the study of alternative control methods, with an emphasis on non-chemical approaches, is increasingly important [2].

These include the development of genetically resistant strains [3], potential contamination of meat, milk, and other by-products intended for human consumption [4], and negative effects on beneficial soil organisms, such as dung beetles [5]. Alternative biological methods, including the use of entomopathogenic organisms, bacteria, and plants, provide a safer and more sustainable solution for tick control [6]. *Rhipicephalus microplus* is the most significant tick species affecting cattle farming [7]. Among these alternatives, entomopathogenic fungi have been considered. These fungi can be commercially produced to serve as biopesticides. Species from the genera *Beauveria*, *Metarhizium*, *Lecanicillium*, and *Isaria* are the most commonly reported fungi with activity against a variety of pests. Additionally, some soil fungi from other taxonomic groups, such as *Arthrobotrys*, not only exhibit nematocidal properties but also demonstrate entomopathogenic activity [8,9]. Recent studies indicate that certain entomopathogenic fungi, specifically those in the genera *Lecanicillium*, *Flavocillium*, and *Purpureocillium*, exhibit antagonistic properties against parasitic nematodes that affect cattle. This antagonism is attributed to compounds released into the liquid culture medium [10,11]. There have been reports on the entomopathogenic activity of certain nematophagous fungi. For example, *Duddingtonia flagrans* has been observed attacking the flies of the species *Hematobia irritans* [12] and also acting against ticks of the species *Amblyomma cajennense* [13]. Additionally, the nematophagous fungus *Purpureocillium lilacinum* has demonstrated activity against house flies [14], while *Arthrobotrys entomophaga* has been found to act against springtails [15].

These and other nematophagous fungi could be active against *R. microplus* ticks and could be potential candidates for use as a biotechnological alternative for tick control; however, they have not been evaluated to date. This study aimed to evaluate the in vitro effectiveness of liquid culture filtrates from selected strains of the nematophagous fungi *Arthrobotrys musiformis*, *P. lilacinum*, *F. subprimulinum*, and *L. psalliotae* against the tick *R. microplus*. Additionally, it assessed the potential of these filtrates to inhibit oviposition, seeking a sustainable alternative for tick control.

## 2. Materials and Methods

### 2.1. Location

The evaluation of culture filtrates was carried out at the Northeast Regional Research Center, Las Huastecas Experimental Field, Tampico-Mante Highway Km 55, 89610, Villa Cuauhtémoc, Altamira, Tamaulipas, Mexico. The isolation and identification of nematophagous fungi, as well as the production of liquid filtrates, were carried out at the Helminthology Laboratory of CENID-SAI, INIFAP, in Jiutepec, Morelos, Mexico.

### 2.2. Nematophagous Fungi

**Figure 1:**
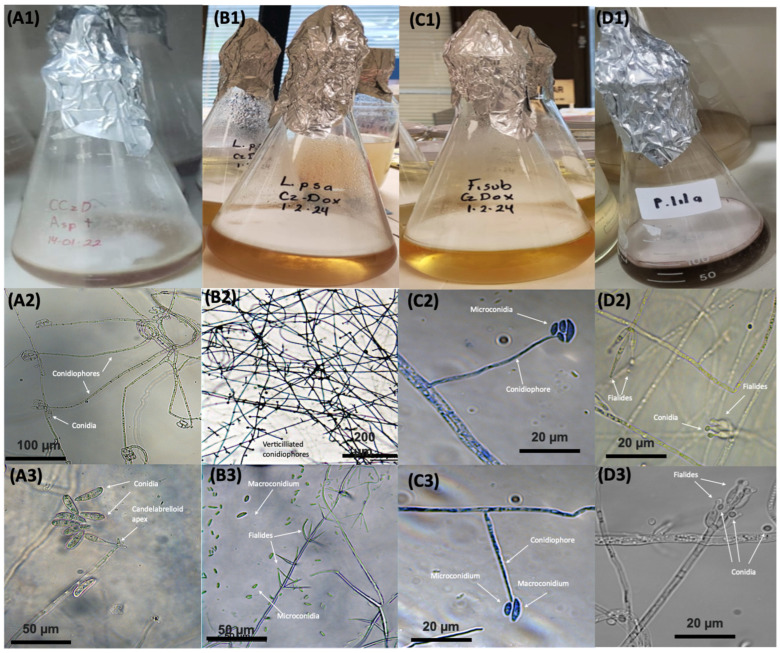
Macroscopic and microscopic characteristics of various fungal strains for obtaining liquid culture filtrates. (**A1**,**B1**,**C1**,**D1**) Macroscopic aspect of four nematophagous fungi: *Arthrobotrys musiformis*, *Lecanicillium psalliotae*, *Flavocillium subprimulinum* and *Purpureocillum lilacinum* growing in flasks containing Czapek-Dox broth medium; (**A2**,**A3**) conidiophores and conidia of *A. musiformis*; (**B2**,**B3**) verticillated conidiophores, fialides and macro and microconidia of *L. psalliotae*; (**C2**,**C3**) conidiophores and macroconidia of *F. subprimulinum*; (**D2**,**D3**) conidia and fialides of *P. lilacinum*.

Four strains of nematophagous fungi from the Helminthology laboratory’s fungal collection were chosen for this study. These strains were chosen based on their previously observed antagonistic activity against nematodes. The selected fungi belong to the following species: *A. musiformis*, *L. psalliotae*, *F. subprimulinum*, and *P. lilacinum* (Figure 1).

The details regarding the fungi, including the isolation source, location, and GenBank Accession number, are presented in Table 1.

### 2.3. Sample Processing for Fungal Isolation

Fungal isolation was conducted using the water-agar plate sprinkling technique, enhanced with nematodes, as described by Barron (1977) [16]. In brief, the plates, sprinkled with the samples, were added with a few drops of an aqueous suspension containing an indeterminate number of free-living nematodes of the species *Panagrellus redivivus*. This nematode species serves as a morphogenesis-inducing agent, stimulating the formation of capture organs and aiding in the identification of the aerial structures of the fungi. After identifying the aerial structures using a stereomicroscope, monoconidial passages were performed. This involved using a fine metal needle to transfer spores to sterile agar water plates for cleaning and purification. The taxonomic identification of the fungi was conducted with the assistance of specialized morphological keys [17,18,19,20,21]. The morphometric taxonomic characterization of the isolates was supported by molecular tests that utilized conserved DNA regions established in our laboratory [22,23,24].

### 2.4. Ticks

The engorged female ticks of *Rhipicephalus microplus* were randomly collected from cattle in an extensive cow-calf production unit in Soto la Marina, Tamaulipas, Mexico. This region is situated on the Gulf of Mexico and has a temperature range of 20 to 26 °C, with altitudes ranging from 50 to 1100 m above sea level. The area experiences an annual rainfall of 700 to 1100 mm and has a semi-warm sub-humid climate characterized by summer rains [25].

The samples were transported to the laboratory in labelled plastic Petri dishes. They were classified by biological stage, including larva, nymph, semi-engorged adult, and engorged adult. Engorged adult ticks were specifically utilized for the bioassays.

### 2.5. Assessment of R. microplus Tick Mortality Using the Immersion Technique

This test involves placing engorged adult ticks in a solution containing a chemical ixodicide or a natural compound derived from a plant or organism, such as nematophagous fungi. In this study, the immersion technique was performed using filtrates from various filamentous fungi cultivated for 4 weeks in 500 mL flasks containing Czapek-Dox culture broth medium (Sigma-Merck, Darmstadt, Germany) at a laboratory temperature of 18–25 °C. The following treatments were designated: (1) *A. musiformis*, (2) *P. lilacinum*, (3) *F. subprimulinum*, (4) *L. psalliotae*, (5) ixodicide mixture (MI; permethrin 5% + chlorpyrifos 24% (LAPISA, La Piedad Michoacán, Mexico) diluted to 1 mL/L) as a positive control, (6) water (negative control) and (7) Czapek-Dox Broth (without fungi) as a negative control. Each treatment was prepared in a total volume of 50 mL of water (H_2_O), to which 300 mg of liquid fungal culture filtrate was added. Ten adult ticks were randomly placed on each plate, with three plates per treatment, and the plates were maintained in a humid chamber. Every treatment was replicated three times to ensure consistency. Adult ticks were immersed in each treatment and incubated in a polypropylene cooler at 28 ± 2 °C. Tick mortality was recorded daily from the start of the experiment and for 20 days post-application (DAA).

### 2.6. Assessment of the Reduction in the R. microplus Oviposition Rate Exposed to Four Fungal Liquid Culture Filtrates

This experiment used the same adult ticks from the assessment described above. The number of ticks that laid eggs was also recorded using the same immersion technique. The procedure for using fungal liquid culture filtrates and the grouping of experimental subjects were the same as those established for tick mortality. This process enabled monitoring of oviposition capacity over 20 days to assess the effect of exposure to fungal filtrates [26,27].

### 2.7. Statistical Analysis

Mortality results were analysed by the Kaplan–Meier method, generating survival curves, and subsequently, the treatment groups were compared using the log-rank test, with the event of interest being the death of the ticks, which was verified by passing a dissecting forceps over each tick and evaluating its reaction and mobility to the stimulus. A factorial ANOVA was also conducted, with mortality as the dependent variable. Treatment (T) and DDA were included as factors. Differences between means were evaluated using Tukey’s test.

Regarding the adult tick oviposition data, they were analysed using a factorial ANOVA design. Oviposition served as the dependent variable, while treatment (T) and DDA were the main factors considered. Mean differences were compared with Tukey’s test, and first-order interactions were evaluated. The Shapiro-Wilk test was employed to confirm that the data follow a normal distribution. The analyses were performed with Statgraphics software, version 18 (2017) [28]. 

## 3. Results

Table 2 presents the results of the ANOVA analysis on tick mortality, evaluating the in vitro lethal effects of liquid culture filtrates from various fungi, an ixodicide mixture, and two control groups. The liquid culture filtrates of *A. musiformis* and *L. psalliotae* caused mortality rates of 40% and 33.3%. against *R. microplus* adult ticks, respectively. Similarly, the ixodicide mixture (Garraban) also resulted in 33.3% tick mortality. In contrast, the control groups using water and Czapek-Dox broth showed lower mortality rates of 16.7% and 13.3%, respectively.

The mortality results for ticks exposed to different filtrates revealed noteworthy findings starting on day 10 of the experiment, particularly when compared with the control group in water. The filtrates from two nematophagous fungi, *L. psalliotae* and *A. musiformis*, demonstrated a significant effect on tick mortality. Specifically, the filtrate of *A. musiformis* showed a consistent increase in effectiveness, reaching 40% tick mortality by days 14, 16, and 18. In comparison, the filtrate of *L. psalliotae* achieved a 33.3% mortality rate during the same period (*p* < 0.01).

It’s important to note that the mortality rate observed with the ixodicidal compound combination (Chlorpyrifos–Permethrin mixture) against ticks began at approximately 6.67% during the first week of the experiment. This rate gradually increased over the following days, reaching a maximum mortality of 33.3% by day 20 (*p* < 0.01).

It is noteworthy that the maximum tick-killing activity achieved by the combination of ixodicidal compounds was still lower than that of the *L. psalliotae* filtrate during the same days of the experiment. Additionally, the filtrates from two of the tested fungi, *P. lilacinum* and *F. subprimulinum*, did not demonstrate any tick-killing activity throughout the experiment.

**Figure 2:**
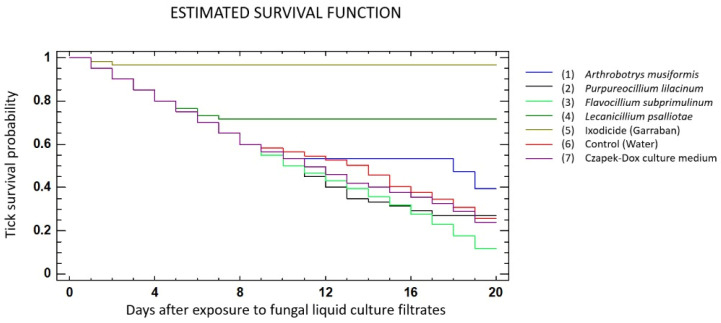
Results about the Kaplan–Meier analysis of the survival probability of *Rhipicephalus microplus* ticks exposed to culture filtrates from four nematophagous fungi.

Kaplan–Meier analysis revealed significant differences in survival estimates between treatments (*p* < 0.01). *Flavocillium subprimulinum* demonstrated the highest probability of survival, while ixodicide, *A. musiformis* and *L. psalliotae* showed the lowest (Figure 2).

With respect to the results of the cumulative risk function on the effect of filtrates from nematophagous fungal cultures on *R. microplus* ticks, the lowest cumulative risk was observed for the ixodicide treatment and the highest effect for *F. subprimulinum*.

Table 3 presents the mean survival time results from the Kaplan–Meier analysis, with the highest being for ixodicide at 19.3 days and the lowest for *Flavocillium subprimulinum* at 11.0 days.

### Oviposition of Adult Ticks

The oviposition of adult ticks was monitored during each sampling period up to 20 days following the application. Treatments using *A. musiformis* culture filtrates and a mixture of two ixodicides (MI) resulted in lower oviposition rates than the control group, starting on the 3rd day. There was a significant reduction following the ixodicide treatment, with an average of 69.8% (*p* < 0.05).

Table 4 presents the effects of liquid culture filtrates from the four studied fungi on the oviposition rates of adult *R. microplus* ticks. Except for the group treated with a mixture of ixodicides, which exhibited a slight reduction, all ticks exposed to the filtrates maintained high oviposition rates (93–100%) (*p* < 0.05).

## 4. Discussion

Acari (including ticks) are exposed to many natural enemies that act as bio-regulators of their populations in their micro-environment. In this way, they have to overcome an intense biological fighting against a wide variety of natural antagonists, including bacteria, viruses, nematodes, protozoa, and a group of micro-fungi called entomopathogenic fungi [29,30,31]; However, some isolates of another group of micro-fungi known as nematophagous fungi that act as parasitic or predators of nematodes in nature, has also been found acting against members of other taxonomic groups in addition to ticks. Such is the case for species in the genus *Arthrobotrys* that have been found attacking flies [9], copepods [32], springtails [33], and even ticks [34]. It is interesting that two groups of micro-fungi with specific highly different blanks of attack, i.e., entomopathogenic fungi that invade and consume insects, and nematophagous fungi that use nematodes as a blank of attack to obtain their nutrients to survive; both groups possesses an enormous wide plasticity, and some isolates are capable to modify their physiological, metabolic, and feeding behavior and eventually taking their nitrogen and carbon sources from insects, arthropods or nematodes [35]. The results obtained in the present study show a clear, although moderate (up to 40%) lethal effect attributed to the liquid culture filtrates obtained from two nematophagous fungi *A. musiformis* and *L. psalliotae* against *R. microplus* adult ticks. In a study performed in Iran, different strains of *Metarhizium anisopliae*, *Beauveria bassiana* and *L. psalliotae* were assessed to investigate their lethal effect against *R. annulatus* engorged females, mortality rates ranging between 90–100% were recorded. Authors report that *L. psalliotae* occasioned a tick mortality of 56.6% and they mentioned that most of strains were able to inhibit the egg laying into a range from 0-26% [36].

Jamra et al. (2024) [37] conducted a study in India to evaluate the effectiveness of the entomopathogenic fungi *Beauveria bassiana* and *M. anisopliae* for controlling *Rhipicephalus microplus* ticks. They used engorged adult ticks in an immersion test and found that treatment with *B. bassiana* resulted in a 19.4% mortality rate among adult female ticks, along with a 44.7% reduction in oviposition. In contrast, ticks treated with *M. anisopliae* displayed the lowest reproductive index (0.56) and the highest level of oviposition inhibition (7.9%). Additionally, the lowest hatching rate (73.4%) was observed in the eggs laid by ticks that received either *B. bassiana* or *M. anisopliae* treatment. These findings are consistent with the results of the present study, which also highlighted the impact of these treatments on oviposition, and mortality in ticks. Therefore, these biological control methods could be valuable additions to integrated tick management programs. Premoli-Azevedo et al. (2015) [34] conducted a study in Brazil to examine the effect of the nematophagous fungus *Pochonia chlamydosporia* on the tick *R. microplus* using engorged female ticks. The ticks were divided into three treatment groups, each immersed in solutions containing 5000, 10,000, and 15,000 chlamydospores, along with a control group. The authors reported that the fungus was able to infect and consume the ticks at all tested concentrations. Similar to the findings of the present study, *P. chlamydosporia* promises to be an alternative method for the biological control of the tick *R. microplus*. Other fungi classified into the order Hypocreales, i.e., *Fusarium beomiforme*, have been recently investigated in India as a potential bio-control agent of *R. microplus* and 100% mortality against adult females and nymphs, as well as 100% reduction in the egg laying capacity, were recorded at 1 × 10^8^ spore concentration [38]. Several entomopathogenic fungi have demonstrated significant activity against adults, nymphs, and eggs of various tick species. For example, *M. anisopliae* has exhibited strong lethal effects on *Ixodes ricinus*, *Amblyomma cajennense*, and *R. microplus* [39,40,41,42]. Other fungal species, such as *Alternaria* sp., *Aspergillus* sp., and *Fusarium* sp., have also caused substantial mortality in both adult and larval *Hyalomma* spp. ticks [43].

These findings provide robust confidence in using selected fungi as biotechnological and sustainable tools of control of *R. microplus* ticks. In another study, the liquid culture filtrates of *A. musiformis* and *A. oligospora* were recently assessed to investigate their potential in vitro lethal activity against infective larvae of the hematophagous parasitic nematode of small ruminants *Haemonchus contortus*, obtaining 91.4% and 86.2% larval mortality, respectively, at 100 mg/mL [44]. Likewise, *L. psalliotae* liquid culture filtrates showed 97.2% ovicidal activity at 24 mg/mL concentration [22].

Regarding the combination of chemical ixodicide drugs composed by Chlorpyrifos–Permethrin used in the present study, showed a lower ixodicide effect against adult ticks (<10%). This suggests that the *R. microplus* strain we used may have evolved a detoxification mechanism against these two drugs through mutations that confer such low efficiency [45]. Likewise, these drugs didn’t show an important oviposition inhibition in the ticks of the present study. The establishment of ixodicide resistance in ticks is a worrying and increasing problem that makes the drugs every time more and more ineffective. In a recent publication performed in Egypt, Deltametrine and Cypermetryn showed an ovicidal activity up to 82 and 60%, respectively, in the egg hatching inhibition against *R. annulatus* eggs [46]. In another study performed in Honduras assessing oviposition inhibition in *R. microplus* ticks, 30% efficacy was reported with Cypermethrin [47]. Research on the antagonistic activity of nematophagous fungi against insects remains limited. Notably, some entomopathogenic fungi are known to produce secondary metabolites—such as beauvericin, beauverolides, bassianolide, and destruxins—that disrupt tick physiology and contribute to pest control [48]. In the present study, specific nematophagous fungi released compounds with acaricidal properties into the culture medium. The identification and characterization of these compounds, preferably using chromatographic techniques, could facilitate the development of natural alternatives to synthetic acaricides. Nevertheless, further research is essential, including comprehensive chemical characterization as well as toxicity and safety evaluations, to confirm that these compounds are both effective and safe for humans, animals, and the environment [49]. The disadvantages of using chemical insecticides are significant. These include the development of pest resistance, environmental contamination, and potential risks to public health from insecticide residues in meat, milk, and other animal products intended for human consumption. To address these issues, it is essential to explore sustainable control alternatives, such as nematophagous fungi and entomopathogenic fungi.

## 5. Conclusions

This study demonstrated that liquid culture filtrates from two nematophagous fungi, *Arthrobotrys musiformis* and *Lecanicillium psaliotae*, significantly affected the mortality of adult ticks of the species *Rhipicephalus* (*Boophilus*) *microplus*. These fungi could be promising candidates for future research to evaluate their potential as control agents for this important ectoparasite, which poses a severe threat to livestock globally.

## Figures and Tables

**Table 1:** Description of the strains used in this study; GenBank accession number from the ITS region for each strain.

Strain	Isolation Source	Location	GenBank Accession Number
*Arthrobotrys musiformis*	Garden soil	Cuautla, Morelos	PP333206
*Lecanicillium psalliotae*	Fig roots and soil	Tepalcingo, Morelos	PX471307
*Flavocillium subprimulinum*	Agricultural soil	Tetela del Volcán, Morelos	PX471371
*Purpureocillum lilacinum*	Leaf litter	Campeche, Campeche	MT052371

**Table 2:** Tick mortality (%) in vitro lethal effects of liquid culture filtrates from four nematophagous fungi against adult *Rhipicephalus microplus* ticks.

FACTOR	P	*n*	Mean ± SE	Tukey Test	DBM	HPMME
**TREATMENT**	**					
1 *Arthrobotrys musiformis*		60	21.16 ± 1.21	a	10	40.0
2 *Purpureocillum lilacinum*		60	8.00 ± 1.21	b	14	16.7
3 *Flavocillium subprimulinum*		60	7.50 ± 1.21	b	11	10.0
4 *Lecanicillium psalliotae*		60	21.00 ± 1.21	a	5	33.3
5 Ixodicide (Garraban)		60	24.33 ± 1.21	a	1	33.3
6 Control (Water)		60	11.67 ± 1.21	b	9	16.7
7 Czapek-Dox culture medium		60	10.83 ± 1.21	b	9	13.3
**(DDA)**	**				**TMM**	
1		21	0.95 ± 2.05	e	Ix	6.7
2		21	0.95 ± 2.05	e	Ix	6.7
3		21	1.43 ± 2.05	e	Ix	6.7
4		21	1.43 ± 2.05	e	Ix	6.7
5		21	1.90 ± 2.05	de	Ix	10.0
6		21	1.90 ± 2.05	de	Ix	10.0
7		21	3.33 ± 2.05	de	Ix	10.0
8		21	3.81 ± 2.05	cde	Ix	13.3
9		21	8.57 ± 2.05	cde	Ix	23.3
10		21	11.90 ± 2.05	bcd	Am	26.7
11		21	13.81 ± 2.05	bc	Am	30.0
12		21	13.81 ± 2.05	bc	Am	30.0
13		21	13.81 ± 2.05	bc	Am	30.0
14		21	20.95 ± 2.05	b	Am	40.0
15		21	20.95 ± 2.05	b	Am	40.0
16		21	19.05 ± 2.05	b	Am	40.0
17		21	19.05 ± 2.05	b	Am	40.0
18		21	20.48 ± 2.05	b	Am	40.0
19		21	20.48 ± 2.05	b	Am	40.0
20		21	100.00 ± 2.05	a	Am	100.0
		**420**	**14.93**			

Different literals express significant differences between treatments (*p* < 0.05); DDA: days post-application; DBM: day of the beginning of mortality; TMM: treatment that showed maximum mortality; HPMME: higher mortality shown during the experiment. Ix: ixodicide; Am: *Arthrobotrys musiformis*; P: probability level (significance) ** *p* < 0.01. Note: The variability.

**Table 3:** Mean survival time of adult *Rhipicephalus microplus* ticks treated with liquid culture filtrates of four nematophagous fungi.

Treatment	Mean Survival Time ± SE
1 *Arthrobotrys musiformis*	12.88 ± 0.94
2 *Purpureocillum lilacinum*	11.20 ± 0.83
3 *Flavocillium subprimulinum*	11.02 ± 0.76
4 *Lecanicillium psalliotae*	15.29 ± 0.82
5 Ixodicide (Garraban)	19.37 ± 0.0
6 Control (Water)	12.08 ± 0.82
7 Czapek-Dox culture medium	11.66 ± 0.77

SE: standard error.

**Table 4:** Oviposition (%) of adult ticks *Rhipicephalus microplus*, effects of liquid culture filtrates from four nematophagous fungi.

FACTOR	P	*n*	Mean ± SE	Tukey Test	HOSDE
**TREATMENT**	**				
1 *Arthrobotrys musiformis*		60	88.00 ± 1.31	ab	96.7
2 *Purpureocillum lilacinum*		60	89.33 ± 1.31	ab	100.0
3 *Flavocillium subprimulinum*		60	91.67 ± 1.31	a	100.0
4 *Lecanicillium psalliotae*		60	85.67 ± 1.31	b	93.3
5 Ixodicide (Garraban)		60	69.83 ± 1.31	c	83.3
6 Control (Water)		60	84.33 ± 1.31	b	96.7
7 Czapek-Dox culture medium		60	85.50 ± 1.31	b	96.7
**(DDA)**	**				
1		21	0.00 ± 2.21	d	0.0
2		21	36.67 ± 2.21	c	60.0
3		21	73.81 ± 2.21	b	96.7
4		21	73.81 ± 2.21	b	96.7
5		21	91.90 ± 2.21	a	100.0
6		21	91.90 ± 2.21	a	100.0
7		21	94.29 ± 2.21	a	100.0
8		21	94.29 ± 2.21	a	100.0
9		21	94.29 ± 2.21	a	100.0
10		21	94.76 ± 2.21	a	100.0
11		21	95.24 ± 2.21	a	100.0
12		21	95.24 ± 2.21	a	100.0
13		21	95.24 ± 2.21	a	100.0
14		21	95.24 ± 2.21	a	100.0
15		21	95.24 ± 2.21	a	100.0
16		21	95.24 ± 2.21	a	100.0
17		21	95.24 ± 2.21	a	100.0
18		21	95.24 ± 2.21	a	100.0
19		21	95.24 ± 2.21	a	100.0
20		21	95.24 ± 2.21	a	100.0
		**420**	**84.90**		

Different literal expresses significant differences between treatments (*p* < 0.05); HOSDE: higher oviposition shown during the experiment. P: probability level (significance) ** *p* < 0.01.

## Data Availability

The original contributions presented in this study are included in the article. Further inquiries can be directed to the corresponding authors.
